# Functional Mitral Regurgitation Post-Isolated Aortic Valve Replacement

**DOI:** 10.3390/jcm13226971

**Published:** 2024-11-19

**Authors:** Petar Dabic, Bojan Vucurevic, Milorad Sevkovic, Dusan Andric, Slobodan Pesic, Mihailo Neskovic, Sasa Borovic, Jovan Petrovic

**Affiliations:** 1 Department of Cardiology and Internal Medicine, Vascular Surgery Clinic, Institute for Cardiovascular Diseases Dedinje, 11000 Belgrade, Serbia; 2 Department of Cardiology, University Children’s Hospital, 11000 Belgrade, Serbia; 3 Vascular Surgery Clinic, Institute for Cardiovascular Diseases Dedinje, 11000 Belgrade, Serbia; 4 Faculty of Medicine, University of Belgrade, 11000 Belgrade, Serbia; sborovic@ikvbd.com; 5 Heart Failure Center, Institute for Cardiovascular Diseases Dedinje, 11000 Belgrade, Serbia

**Keywords:** patient-prosthesis mismatch, aortic valve replacement, mitral regurgitation, aortic stenosis

## Abstract

**Background**: The management of mitral regurgitation during aortic valve replacement remains a complex question. Secondary mitral regurgitation often improves post-aortic valve replacement without mitral valve surgery, but residual mitral regurgitation can significantly affect long-term outcomes. This study investigates the natural history of mitral regurgitation following isolated aortic valve replacement and identifies prognostic factors for persistent mitral regurgitation. **Methods**: A retrospective study was conducted on 108 patients who underwent isolated aortic valve replacement. Patients were categorized based on mitral regurgitation improvement. Additionally, patients were divided into patient-prosthesis mismatch and non-patient-prosthesis mismatch groups based on the aortic prosthesis. Preoperative and postoperative echocardiographic data were analyzed. **Results**: In total, 63% of patients showed mitral regurgitation improvement. The improved functional MR group showed significant reductions in peak and mean transvalvular pressure gradients. In contrast, the patient-prosthesis mismatch group had persistent mitral regurgitation improvement in 59.2% of patients. The non-patient-prosthesis mismatch group exhibited significant structural improvements and a reduction in mitral regurgitation severity in 68.6% of patients. **Conclusions**: The study shows that aortic valve replacement could significantly improve MR when patient-prosthesis mismatch is avoided. This approach maximizes hemodynamic outcomes, mitigates the risk of residual or worsening mitral regurgitation, and potentially reduces the need for additional mitral valve interventions.

## 1. Introduction

Mitral regurgitation (MR) accompanies severe aortic valve stenosis (AS). Around 75% of patients undergoing aortic valve replacement (AVR) for AS have some degree of MR [1,2]. The management of MR in patients undergoing AVR remains a complex and debated area in cardiovascular surgery, particularly when considering whether to perform concomitant MV interventions. The variability in guidelines reflects differing interpretations of the available evidence and the challenges of balancing potential benefits against increased surgical risks [3,4].

Surgical intervention on the mitral valve (MV) is generally unnecessary when there are no leaflet abnormalities, annulus distention, or significant left ventricular (LV) geometry issues. Additionally, non-severe secondary MR frequently betters following aortic valve treatment [3]. While MV surgery during AVR increases perioperative complications, the effect of residual MR on survival, quality of life, and myocardial remodeling is significant. Perioperative mortality rates following AVR are around 2.2%. However, in patients undergoing both AVR and MV replacement, these rates rise to 9% [4]. Several studies suggest that MV repair during double valve surgery might be advantageous compared to MV replacement, potentially reducing long-term risk without a corresponding rise in perioperative mortality [5,6,7]. According to some studies, patients whose MR improved postoperatively had significantly higher 5-year survival rates compared to those without MR improvement [8,9].

AVR for severe AS is designed to decrease LV afterload, potentially initiating reverse remodeling of the LV. These changes are anticipated to positively influence MV mechanics, potentially resolving secondary MR that arises without structural abnormalities of the mitral apparatus. However, the expected improvement in MR is often not realized.

This study aimed to investigate the natural progression of functional MR (FMR) following AVR and identify echocardiographic parameters linked to persistent MR.

## 2. Materials and Methods

In continuation of our earlier published research, we conducted a case-control study with an expanded sample size [10]. Over 16 years, 3014 patients underwent isolated AVR for severe AS in our tertiary care center. We included adult patients, aged 18 and older, who had undergone aortic valve replacement with either a bileaflet mechanical or bioprosthetic valve and who had FMR. Eligible patients required postoperative echocardiographic data at both discharge and at a 6-month follow-up to assess outcomes over time. Only patients with stable postoperative conditions, free from complications necessitating additional surgery were included. Preoperative echocardiographic data, such as left ventricular outflow tract diameter and left ventricular ejection fraction, were required for baseline comparisons. Exclusion criteria included morphological mitral apparatus abnormalities, chordal rupture, leaflet calcification, fibrosis or prolapse, coronary artery disease, aortic disease, previous open-heart procedures, and congenital heart abnormalities. Ultimately, 108 patients were included in our study, which were analyzed retrospectively. AVR was performed using St. Jude Medical™ Hemodynamic Plus Aortic Valve and St. Jude Medical™ Biocor™ Pericardial Stented Tissue Valve.

Patients were categorized into two groups based on the presence of improvement in their FMR post-AVR. The Persistent FMR group maintained a moderate to moderate-severe grade (2+ and 3+) of MR after AVR. The Improved FMR group showed a reduction in MR grade (less than 2+) following AVR. The MR severity was based on the measurement of vena contracta. Additionally, patients were divided based on the indexed Effective Orifice Area (EOAi) of the implanted aortic prosthesis. The EOA was calculated using the manufacturer’s published values, which were then indexed to the body surface area. The Prosthesis-Patient Mismatch group (PPM) included patients with an EOAi ≤ 0.85 cm^2^/m^2^, and the Non-Prosthesis-Patient Mismatch group (non-PPM) included patients with an EOAi > 0.85 cm^2^/m^2^.

All patients included in the study underwent preoperative and postoperative transthoracic echocardiography. Postoperative echocardiography was routinely performed at discharge and again at the 6-month follow-up. For analysis, we used data from the 6-month follow-up assessment. The echocardiographic analysis included a comprehensive assessment using M-mode, two-dimensional imaging, and Doppler echocardiography protocols as per the guidelines set forth by the European Association of Cardiovascular Imaging and the American Society of Echocardiography [11,12,13]. All patients provided written informed consent for the publication of their study data.

### Statistical Analysis

We conducted data analysis using parametric or nonparametric methods based on the nature of the variables. Descriptive statistics were used to express observed characteristics, including mean values with standard deviation for normally distributed data, and median with interquartile range for non-normally distributed data. For continuous nonparametric data, Wilcoxon’s signed-rank test was employed, whereas continuous parametric data were analyzed using Student’s *t*-test and paired *t*-test as appropriate. Categorical data were analyzed using the Chi-square test or Fisher’s exact test to determine statistically significant differences between groups. The significance level was set at 2-sided *p* < 0.05. The statistical analysis was performed using SPSS Statistics 26 (IBM, Armonk, NY, USA).

## 3. Results

We observed that 68 patients (62.9%) showed improvement in FMR postoperatively, while 40 patients (37.1%) had persistent FMR after the procedure. There were no significant differences in preoperative parameters, except for the value of left ventricular ejection fraction (LVEF) between persistent and improved FMR groups (40.6 ± 18.1% vs. 54.3 ± 12.9%, *p* = 0.028) (Table 1).

A significant reverse remodeling of the LV was observed evidenced by reductions in LV end-diastolic diameter (LVEDD), LV end-systolic diameter (LVESD), LV posterior wall and septum thickness (Table 2). These findings collectively indicate favorable changes in LV structure and function following AVR in patients with improved FMR.

The PPM group included 22 patients (20.4%) and the non-PPM included 86 patients (79.6%) (Table 3). The average EOAi for the PPM group was 0.72 ± 0.61 and for non-PPM 1.11 ± 0.20 cm^2^/m^2^. In the PPM group following AVR, there was a notable decrease in transvalvular pressure gradients. Despite these reductions, other echocardiographic parameters remained largely unchanged. Notably, MR grade persisted at ≥2+ in 59.2% of the patients, indicating that while AVR effectively reduced pressure gradients, it did not uniformly improve MR severity in patients with PPM (Table 4). Conversely, in the non-PPM group, following AVR, significant reductions were also observed in both transvalvular pressure gradients (Table 4). Additionally, significant reverse remodeling of the LV occurred, evidenced by reductions in LVEDD, LVESD, septum thickness, and LV posterior wall thickness. This indicates a favorable structural and functional adaptation of the LV in this group. Furthermore, a substantial improvement in MR was noted, with the MR grade reducing below 2+ in most patients (68.6%) (Table 4). This underscores the more consistent and beneficial effects of AVR in the non-PPM group in terms of both pressure gradient reduction and MV function.

## 4. Discussion

The data collected from our study provide critical insights into the differential impacts of AVR on patients with and without PPM. The impact of AVR on MR and other echocardiographic parameters shows notable differences between the PPM and non-PPM groups.

These differences may be due to several factors. First, the persistence of MR post-AVR suggests that factors other than LV afterload may play a role in the pathophysiology of MR in these patients. For instance, the duration and severity of AS before AVR might lead to irreversible changes in the LV that continue to affect MV function even after the correction of AS. Moreover, the presence of PPM is another critical variable influencing outcomes. As observed in our study, patients with PPM showed less improvement in MR post-AVR compared to those without mismatch. This suggests that optimal prosthesis sizing and selection are crucial for maximizing the therapeutic benefits of AVR, including the mitigation of secondary MR.

Barreiro et al. observed significant postoperative improvements in MR, with 82% of patients showing resolution [9]. This high rate of improvement underscores the potential for significant cardiac function recovery when left ventricular afterload is reduced by AVR. Similarly, Vanden Eynden et al. highlighted the predictive role of preoperative factors, noting that isolated ischemic and functional MR were significant predictors of MR improvement post-AVR [14]. This insight is particularly valuable as it suggests that the nature of MR, whether ischemic or functional, can influence the therapeutic outcomes of AVR, emphasizing the need for a nuanced preoperative assessment. In our study, the improvement rate of FMR postoperatively was 62.9%. While this rate is somewhat lower than that reported by Barreiro et al., it nonetheless represents a substantial proportion of patients experiencing beneficial changes in mitral valve function following AVR. The disparity in improvement rates may be attributed to differences in patient populations, severity of preoperative MR, or the presence of factors such as PPM, which we found to influence postoperative outcomes.

On the other hand, Asher et al. demonstrated that MV repair or replacement for more-than-moderate MR at the time of CABG may be reasonable in a suitably selected CABG population but not for AVR, with or without coronary artery bypass grafting [15].

Harling et al. showed that the structural remodeling caused by severe AS regresses after AVR, as evidenced by reductions in LV mass and end-diastolic diameter. [16]. Some studies identified factors associated with LV remodeling, such as higher preoperative LV mass, larger LV diastolic diameter and end-diastolic volume as independent predictors of improvement in MR after aortic valve surgery. These studies suggest that when there is potential for reverse remodeling, a more substantial improvement in MR is likely to occur after AVR [17,18]. Our study demonstrated an association between improvements in FMR and markers of LV remodeling, as shown by reductions in LVEDD, LVESD, and septal and LV posterior wall thickness. However, we recognize that our findings are associative and do not establish a causal relationship between FMR improvement and LV remodeling.

The findings of Harling et al. and subsequent research underscore a key physiological insight: the structural changes in the heart due to severe AS are not permanent and can be partially reversed following AVR. This process of reverse remodeling includes reductions in LV mass, LVEDD, and other structural dimensions, which are vital for improving cardiac function and patient outcomes [16,17,18]. A reduction in the size and mass of the LV generally leads to less tension on the MV apparatus, thereby improving leaflet coaptation and reducing regurgitation. Additionally, a decrease in the pressure and volume overload in the LV due to improved valve function helps in normalizing the dimensions and functioning of the heart, which contributes to the alleviation of MR [19].

Several studies identified factors associated with the progression of MR [20,21]. These included left atrial growth, atrial fibrillation, LV dysfunction, peak AV gradient < 60 mmHg, increased LV mass, elevated tricuspid regurgitation (TR) velocity, and elevated LV mass. In contrast, Joo et al. reported that increased right ventricular systolic pressure was the only significant predictor of postoperative MR [22]. Unger et al. found that postoperative MR was more likely to improve in patients with reduced LVEF and increased LV size [17]. Jeong et al. demonstrated that patients with preoperative atrial fibrillation and an LVEF > 40% were more likely to have residual MR [23]. Additionally, Sehovic et al. found that LVEDD > 54 mm, effective regurgitant orifice area > 25 mm^2^, regurgitation volume > 40 mL/beat, pulmonary artery systolic pressure > 40 mmHg, and LA diameter > 45 mm were factors contributing to worsening MR [24]. PPM and low LVEF are both significant predictors of limited FMR recovery post-AVR.

In patients with moderate MR undergoing AVR, particularly those with low LVEF, we recognize that limited improvement in LVEF and the potential for ongoing LV and annular dilatation may restrict LV remodeling and FMR improvement. This raises a clinically relevant consideration for concomitant MVR. Moreover, PPM can place an additional and continuous load on the LV, which could impede LV remodeling and FMR recovery.

The findings in our study were the significant differences in postoperative pressure gradients between the persistent and improved FMR groups. Specifically, we observed substantial reductions in both peak and mean transvalvular gradients postoperatively. In the persistent FMR group, the postoperative peak gradient reduction was 61.8 mmHg (*p* = 0.002), and the mean gradient reduction was 39.9 mmHg (*p* = 0.001). In contrast, the improved FMR group demonstrated even greater reductions, with a postoperative peak gradient reduction of 79 mmHg (*p* = 0.001) and a mean gradient reduction of 57 mmHg (*p* = 0.003). These findings indicate a robust reduction in transvalvular gradients particularly in the improved FMR group. The lower preoperative values of LVEF observed in the persistent FMR group may partially explain this phenomenon, suggesting a “low flow–low gradient” effect. In this scenario, compromised LV function before surgery can lead to lower flow rates across the aortic valve, resulting in less significant pressure gradients even after AVR.

Previous studies have highlighted the negative impact of more than mild PPM, defined as an EOAi ≤ 0.85 cm^2^/m^2^, on various outcomes following AVR. These outcomes include less symptomatic improvement, worse hemodynamics at rest and during exercise, reduced regression of LV hypertrophy, and increased cardiac events postoperatively [25]. In our study, we specifically investigated the impact of aortic prosthesis size, and thus PPM, on the evolution of FMR. Our findings revealed that patients without PPM experienced not only significant reductions in postoperative peak and mean gradients but also beneficial reverse remodeling of the LV, as evidenced by reductions in LVEDD, LVESD, septum thickness, and LV posterior wall thickness. Furthermore, the improvement in FMR grade below 2+ was notably higher in the non-PPM group (68.6%) compared to the PPM group (59.2%). It is important to note that our study employed an identical model of mechanical and tissue prostheses across all patients, thereby eliminating the potential influence of different manufacturer designs on our results. However, contrasting findings by Waisbren et al. suggested no independent relationship between aortic prosthesis size and changes in MR [26].

### Limitations

While our study benefited from a well-defined and homogeneous FMR group due to restrictive patient selection criteria, the limitation of a small sample size, particularly in comparing the non-PPM and PPM groups (86 vs. 22 patients, respectively), must be acknowledged. Calculating the EOAi based on the manufacturer’s published values, rather than on patient-specific post-surgical measurements, may limit the precision of our findings. This approach was chosen due to limited availability of direct postoperative measurements. The accuracy of EOA echocardiographic measurement in bioprosthetic valves is restricted by similar challenges to those in AVA measurement; the LV outflow tract diameter could be difficult to measure due to reverberation artifacts from the prosthetic valve. For bileaflet mechanical valves, the central orifice could produce a high-velocity jet, leading to potential EOA underestimation. We recognize the possibility that these projected values may overestimate EOAi and the incidence of PPM. Larger studies with more robust patient numbers are needed to validate the relationships between aortic prosthesis size, PPM, and the evolution of FMR following AVR.

## 5. Conclusions

Our study showed an improvement in FMR following AVR surgery in the majority of patients. Our findings also suggest that PPM may adversely affect the reduction of FMR. Optimal prosthesis sizing and selection are crucial for maximizing the therapeutic benefits of AVR, including the mitigation of secondary MR. We advocate selecting a prosthesis of adequate size to optimize hemodynamic outcomes and mitigate the risk of residual or worsening FMR postoperatively. We support a tailored surgical approach that prioritizes optimizing prosthesis size to avoid PPM, thereby potentially improving outcomes and reducing the need for additional MV interventions in this patient population.

## Figures and Tables

**Table 1:** Patient characteristics according to postoperative mitral regurgitation.

	Persistent FMR (*n* = 40)	Improved FMR (*n* = 68)	*p*-Value
Age (years)	58.5 ± 12.8	63.1 ± 11.2	0.053
Male sex (*n*, %)	23 (57.5)	28 (41.2)	0.150
BMI (kg/m^2^)	26.9 ± 3.9	26.7 ± 4.4	0.412
AF	3 (7.5)	8 (11.7)	0.743
NYHA class			
II	37 (92.5)	65 (95.6)	1.000
III	3 (7.5)	3 (4.4)	
TAV	38 (95.0)	63 (92.6)	1.000
AVA (cm^2^)	0.7 ± 0.1	0.6 ± 0.2	0.127
Peak gradient (mmHg)	91.1 ± 30.7	107.1 ± 31.2	0.061
Mean gradient (mmHg)	55.7 ± 21.2	71.6 ± 24.3	0.059
LVEDD (mm)	56.9 ± 12.5	52.3 ± 6.1	0.093
LVESD (mm)	42.3 ± 12.5	36.3 ± 7.2	0.055
Septum thickness (mm)	12.1 ± 1.8	12.7 ± 1.7	0.723
Posterior wall thickness (mm)	11.9 ± 1.6	11.7 ± 1.5	0.785
LA (mm)	42.3 ± 4.5	43.2 ± 4.7	0.436
LVEF (%)	40.6 ± 18.1	54.3 ± 12.9	0.028
TR grade			
0	22 (55)	37 (54.4)	1.000
I	8 (20)	14 (20.6)	
II	10 (25)	17 (25)	
RVSP (mmHg)	46.4 ± 8.3	44.1 ± 11.2	0.517
EOAi (cm^2^/m^2^)	1.3 ± 0.4	1.2 ± 0.1	0.799

AF—atrial fibrillation; AVA—aortic valve area; BMI—body mass index; EOAi—indexed Effective Orifice Area; FMR—functional mitral regurgitation; LA—left atrium; LVEDD—left ventricle end-diastolic diameter; LVEF—left ventricle ejection fraction; LVESD—left ventricle end-systolic diameter; RVSP—right ventricle systolic pressure; TAV—tricuspid aortic valve; TR—tricuspid regurgitation.

**Table 2:** Echocardiographic parameters of patients according to postoperative mitral regurgitation.

	Persistent FMR (*n* = 40)			Improved FMR (*n* = 68)		
	Preoperative	Postoperative	*p*-Value	Preoperative	Postoperative	*p*-Value
Peak gradient (mmHg)	91.1 ± 30.7	29.3 ± 9.1	0.002	107.1 ± 31.2	28.1 ± 7.23	0.001
Mean gradient (mmHg)	55.7 ± 21.2	15.8 ± 5.4	0.001	71.6 ± 24.3	14.6 ± 4.5	0.003
LVEDD (mm)	56.9 ± 12.5	57.7 ± 11.9	0.912	52.3 ± 6.1	50.1 ± 3.2	0.021
LVESD (mm)	42.3 ± 12.5	41.1 ± 13.6	0.712	36.3 ± 7.2	32.2 ± 5.3	0.010
Septum thickness (mm)	12.1 ± 1.8	11.7 ± 2.1	0.373	12.7 ± 1.7	10.9 ± 1.9	0.004
Posterior wall thickness (mm)	11.9 ± 1.6	12.2 ± 1.5	0.223	11.7 ± 1.5	10.7 ± 1.2	0.022
LA (mm)	42.3 ± 4.5	42.3 ± 4.8	0.577	43.2 ± 4.7	42.1 ± 4.1	0.059
LVEF (%)	40.6 ± 18.1	41.8 ± 15.9	0.844	54.3 ± 12.9	57.4 ± 7.5	0.193
TR grade						
0	22 (55)	19 (47.5)	0.527	37 (54.4)	34 (50)	0.527
I	8 (20)	6 (15)		14 (20.6)	22 (32.4)	
II	10 (25)	15 (37.5)		17 (25)	11 (16.1)	
III	0	0		0	1 (1.5)	
RVSP (mmHg)	46.4 ± 8.3	40.8 ± 9.1	0.589	44.1 ± 11.2	39.9 ± 3.2	0.248

AVR—aortic valve replacement; FMR—functional mitral regurgitation; LA—left atrium; LVEDD—left ventricle end-diastolic diameter; LVEF—left ventricle ejection fraction; LVESD—left ventricle end-systolic diameter; RVSP—right ventricle systolic pressure; TR—tricuspid regurgitation.

**Table 3:** Preoperative patients’ characteristics according to Prosthesis-Patient Mismatch.

	PPM (*n* = 22)	Non-PPM (*n* = 86)	*p*-Value
Age (years)	70.1 ± 5.9	68.2 ± 7.1	0.079
Male sex (*n*, %)	6 (27.3)	38 (44.2)	0.224
BMI (kg/m^2^)	25.7 ± 3.9	28.7 ± 5.5	0.681
AF	5 (22.7)	13 (15.1)	0.521
NYHA class			
II	19 (86.7)	81 (94.2)	0.356
III	3 (13.3)	5 (5.8)	
TAV	21 (95.5)	72 (83.7)	0.297
AVA (cm^2^)	0.7 ± 0.4	0.7 ± 0.2	0.513
Peak gradient (mmHg)	89.7 ± 23.1	107.1 ± 30.2	0.472
Mean gradient (mmHg)	55.6 ± 20.1	67.2 ± 26.8	0.516
LVEDD (mm)	54.7 ± 5.2	53.9 ± 9.2	0.881
LVESD (mm)	37.6 ± 5.4	37.1 ± 11.1	0.934
Septum thickness (mm)	11.8 ± 1.9	12.2 ± 1.9	0.764
Posterior wall thickness (mm)	12.1 ± 1.1	11.9 ± 1.9	0.611
LA (mm)	43.3 ± 2.7	41.9 ± 5.3	0.799
LVEF (%)	49.9 ± 14.9	50.2 ± 16.1	0.862
TR grade			
0	16 (72.7)	45 (52.3)	0.064
I	6 (27.3)	27 (31.4)	
II	0	14 (16.3)	

AF—atrial fibrillation; AVA—aortic valve area; BMI—body mass index; LA—left atrium; LVEDD—left ventricle end-diastolic diameter; LVEF—left ventricle ejection fraction; LVESD—left ventricle end-systolic diameter; PPM—Prosthesis-Patient Mismatch; TAV—tricuspid aortic valve; TR—tricuspid regurgitation.

**Table 4:** Echocardiographic parameters of patients according to Prosthesis-Patient Mismatch.

	PPM (*n* = 22)			Non-PPM (*n* = 86)		
	Preoperative	Postoperative	*p*-Value	Preoperative	Postoperative	*p*-Value
Peak gradient (mmHg)	89.7 ± 23.1	32.9 ± 11.7	0.012	107.1 ± 30.2	28.3 ± 7.2	0.008
Mean gradient (mmHg)	55.6 ± 20.1	18.2 ± 7.1	0.027	67.2 ± 26.8	15.1 ± 4.3	0.002
LVEDD (mm)	54.7 ± 5.2	54.1 ± 4.9	0.675	53.9 ± 9.2	52.7 ± 8.8	0.032
LVESD (mm)	37.6 ± 5.4	36.7± 4.9	0.771	37.1 ± 11.1	35.1 ± 11.1	0.010
Septum thickness (mm)	11.8 ± 1.9	11.2 ± 1.5	0.473	12.2 ± 1.9	10.8 ± 1.5	0.001
Posterior wall thickness (mm)	12.1 ± 1.1	11.8± 0.9	0.464	11.9 ± 1.9	10.91 ± 1.49	0.001
LA (mm)	43.3 ± 2.7	42.7 ± 1.6	0.822	41.9 ± 5.3	41.8 ± 4.9	0.221
LVEF (%)	49.9 ± 14.9	51.5 ± 11.1	0.207	50.2 ± 16.1	51.9 ± 14.2	0.287
TR grade						
0	16 (72.7)	11 (50)	0.157	45 (52.3)	40 (46.5)	0.434
I	6 (27.3)	8 (36.4)		27 (31.4)	25 (29.1)	
II	0	3 (13.6)		14 (16.3)	19 (22.1)	
III	0	0		0	2 (2.3)	
MR grade						
≥2+	22 (100)	13 (59.2)	0.002	86 (100)	27 (31.4)	0.000
<2+	0	9 (40.8)		0	59 (68.6)	

AVR—aortic valve replacement; LA—left atrium; LVEDD—left ventricle end-diastolic diameter; LVEF—left ventricle ejection fraction; LVESD—left ventricle end-systolic diameter; MR—mitral regurgitation; PPM—Prosthesis-Patient Mismatch; TR—tricuspid regurgitation.

## Data Availability

The data presented in this study are available on request from the corresponding author due to ethical reasons.
